# Angiogenic factors for planning fetal surveillance in fetal growth restriction and small‐for‐gestational‐age fetuses: A prospective observational study

**DOI:** 10.1111/1471-0528.17151

**Published:** 2022-04-05

**Authors:** Erika Bonacina, Manel Mendoza, Alba Farràs, Pablo Garcia‐Manau, Berta Serrano, Ivan Hurtado, Raquel Ferrer‐Oliveras, Lidia Illan, Mireia Armengol‐Alsina, Elena Carreras

**Affiliations:** ^1^ Maternal Fetal Medicine Unit, Department of Obstetrics Hospital Universitari Vall d'Hebron, Universitat Autonoma de Barcelona Barcelona Spain

**Keywords:** fetal Doppler, fetal growth restriction, fetal surveillance, PlGF, sFlt‐1, small for gestational age

## Abstract

**Objective:**

The aim of this study was to assess the added value of the soluble fms‐like tyrosine kinase‐1 (sFlt‐1) and placental growth factor (PlGF) ratio for adjusting the periodicity of ultrasound examinations in early‐onset fetal growth restriction (FGR) and small for gestational age (SGA).

**Design:**

A prospective, observational study.

**Setting:**

Tertiary referral hospital.

**Population:**

One hundred and thirty‐four single pregnancies with ultrasonographic estimated fetal weight (EFW) below the 10th centile between 20^+0^ and 31^+6^ weeks of gestation with antegrade umbilical artery flow.

**Methods:**

The time from Doppler and sFlt‐1/PlGF assessment to delivery was recorded and classified into four ranges: <1, <2, <3 and <4 weeks.

**Main outcome measures:**

Sensitivity (Sn), specificity (Sp), positive predictive value (PPV) and negative predictive value (NPV) of sFlt‐1/PlGF values to predict the time to delivery.

**Results:**

In the SGA cohort, the NPV calculated for an sFlt‐1/PlGF cut‐off value of 38 was 100% for delivery before 3 weeks, and 98% for delivery before 4 weeks after diagnosis (95% CI 0.89–1.00). In the FGR cohort, the NPV calculated for an sFlt‐1/PlGF cut‐off value of 38 was 100% for delivery before 2 weeks after diagnosis (95% CI 0.92–1.00). By contrast, more than 50% of cases with an sFlt‐1/PlGF value of >85 required an elective delivery before 1 week.

**Conclusions:**

sFlt‐1/PlGF values in early‐onset SGA and FGR are predictive of the time to delivery and could be used for planning fetal surveillance, by reducing the frequency of ultrasound in cases with sFlt‐1/PlGF < 38 and by providing closer follow‐up in cases with sFlt‐1/PlGF >85.

**Tweetable abstract:**

sFlt‐1/PlGF values in early‐onset SGA/FGR could be used in addition to Doppler for planning fetal surveillance.

## Tweetable abstract

sFlt‐1/PlGF values in early‐onset SGA/FGR could be used in addition to Doppler for planning fetal surveillance.

**Linked article**: This article is commented on by Cecilia Villalaín, pp. 1878 in this issue. To view this minicommentary visit https://doi.org/10.1111/1471‐0528.17172

## 1 INTRODUCTION

Small fetuses are usually defined as those with an ultrasound estimated fetal weight (EFW) below the 10th centile.^1^ Fetal growth restriction (FGR) is a failure to achieve biological growth potential and is usually accompanied by fetal/placental Doppler anomalies, whereas fetuses deemed small for gestational age (SGA) are usually considered constitutionally small and have normal Doppler results.^2^, ^3^ Along with pre‐eclampsia (PE), FGR and some SGA might be caused by a degree of placental dysfunction, associated with poorer perinatal outcomes.^4^, ^5^ Adequate surveillance and planned delivery for SGA and FGR fetuses can reduce the rate of stillbirth and improve pregnancy outcomes;^6^ however, optimal management is challenging as it requires multiple ultrasounds for fetal Doppler assessment, which represents a healthcare burden and arouses anxiety and stress in patients. The management of FGR and SGA varies considerably internationally, as there is no clear consensus on the adequate frequency of ultrasound examinations.^7^ In early‐onset cases (<32 weeks of gestation) with antegrade flow in the umbilical artery (UA),^8^ most guidelines advise performing Doppler assessment at least weekly for FGR and every 2 weeks for SGA.^7^

Soluble fms‐like tyrosine kinase‐1 (sFlt‐1) and placental growth factor (PlGF) are angiogenic‐related biomarkers of placental dysfunction.^9^ These markers have been demonstrated to predict adverse maternal and perinatal outcomes in PE,^10^ and correlate positively with both FGR/SGA severity and speed of disease progression, which lead to shorter times to delivery.^11^, ^12^ However, their ability to assist with decision making around the frequency of fetal surveillance has not been investigated.

The aim of this study was to assess the added value of the sFlt‐1/PlGF ratio for adjusting the periodicity of ultrasound examinations in early‐onset FGR and SGA.

## 2 METHODS

This is a prospective observational study carried out at Vall d'Hebron University Hospital, Barcelona, Spain, between July 2017 and July 2019. The local ethics committee (CEIC‐VHIR PR[AMI]349/2016) approved the study protocol. Patients were not actively involved in the research. Single pregnancies with an ultrasonographic estimated fetal weight (EFW) below the 10th centile between 20^+0^ and 31^+6^ weeks of gestation were invited to participate. Women were enrolled after providing their written informed consent. The exclusion criteria were UA absent end‐diastolic flow (AEDF), UA reversed end‐diastolic flow (REDF), abnormal ductus venosus flow, known fetal chromosomal abnormalities, congenital defects, PE and stillbirth at the time of diagnosis. FGR was defined as an EFW below the third centile, or EFW between the third and 10th centiles accompanied by a UA pulsatility index (PI) above the 95th centile, a cerebroplacental ratio below the fifth centile, middle cerebral artery PI below the fifth centile and/or UA PI above the 95th centile, and SGA was defined as an EFW of between the third and 10th centiles with no fetal or maternal Doppler anomalies.^1^, ^13^, ^14^, ^15^ PE was defined as the new onset of high blood pressure (systolic blood pressure ≥ 140 mmHg and/or diastolic blood pressure ≥ 90 mmHg), the worsening of previous high blood pressure added to new‐onset proteinuria (protein/creatinine ratio of >300), the worsening of previous proteinuria, or to at least one of the following signs and symptoms: cerebral or visual symptoms, elevation of liver enzymes to twice the normal concentration, platelet count of <100 000/μl, serum creatinine concentrations of >1.1 mg/dl or pulmonary oedema.^16^

Maternal demographic characteristics and medical history, ultrasound assessment of fetal anatomy, EFW and serum concentrations of PlGF and sFlt‐1 were recorded at diagnosis. EFW was measured by fetal biparietal diameter, head circumference, abdominal circumference and femur length,^17^ and EFW centiles were calculated according to customised birthweight standards for a Spanish population.^18^ PlGF and sFlt‐1 were both measured in pg/ml by means of fully automated Elecsys assays on an electrochemiluminescence immunoassay platform (Cobas E analysers; Roche Diagnostics, Basel, Switzerland). Gestational age (GA) was determined by fetal crown–rump length measurement at 11^+0^–13^+6^ weeks of gestation.^19^

Specialists were not blinded to angiogenic factor levels; nevertheless, the timing and mode of delivery were based in all cases on GA, Doppler findings, conventional visual cardiotocography (CTG) interpretation, and maternal signs and symptoms following the current hospital protocols, regardless of the results of the sFlt‐1/PlGF measurement.^1^ According to that protocol, elective delivery was recommended at >40 weeks of gestation for SGA, >37 weeks of gestation for FGR with antegrade UA flow, >34 weeks of gestation for FGR with AEDF and >30 weeks of gestation for FGR with REDF. CTG indications for elective delivery were fetal heart rate sinusoidal tracing or absent fetal heart rate variability, accompanied by recurrent late decelerations, recurrent variable decelerations or bradycardia.^20^

### 2.1 Statistical analysis

The Strengthening the Reporting of Observational Studies in Epidemiology (STROBE) statement was followed for reporting the results.^21^ Categorical data were reported as frequency and percentage and continuous variables were reported as median and interquartile range (IQR). The time from Doppler and sFlt‐1/PlGF assessment to delivery was recorded and classified into four ranges: <1, <2, <3 and <4 weeks. The association between sFlt‐1/PlGF values above the cut‐off values and time to delivery was investigated by calculating the sensitivity (Sn), specificity (Sp), positive predictive value (PPV) and negative predictive value (NPV). Kaplan–Meier survival curves were constructed for the analysis of time to delivery after the diagnosis of early‐onset FGR for sFlt‐1/PlGF cut‐off values of 38, 85, 110, 201 and 655. The two‐sided statistical significance level was set at *p* < 0.05. The R Commander package in R 2.3‐1 was used for statistical analysis.

## 3 RESULTS

During the study period, 134 consecutive women were invited to take part in the study and all agreed to participate. Among them, 48 (35.8%) had a fetus that was SGA and 86 (64.2%) had FGR. All FGR with abnormal Doppler results were associated with UA increased pulsatility. The median GA at diagnosis of SGA and FGR was 26.5 weeks (IQR 24.0–29.0 weeks) and 25.6 weeks (IQR 23.0–29.3 weeks), respectively. The demographic and clinical characteristics of the studied cohorts are shown in Table 1. The median GA at delivery was 38.0 weeks (IQR 37.0–39.0 weeks) for SGA and 37.0 weeks (IQR 34.2–37.0 weeks) for FGR, with low EFW centile being the most common indication in both groups (61.7% in SGA and 38.4% in FGR). More details regarding pregnancy outcomes are presented in Table 2.

**TABLE 1:** Baseline characteristics of the study population

	SGA (*n* = 48)	FGR (*n* = 86)
Maternal age (years)	33.0 (30.0–35.0)	31.0 (27.0–35.7)
Pre‐pregnancy BMI (kg/m^2^)	23 (20.6–25.3)	24.2 (21.6–27.9)
Smoking	5 (10.4%)	15 (17.4%)
Obstetric history		
FGR in previous pregnancy	9 (18.7%)	7 (8.1%)
SGA in previous pregnancy	1 (2.1%)	3 (3.5%)
Stillbirth in previous pregnancy	1 (2.1%)	1 (1.2%)
Previous pre‐eclampsia	4 (8.3%)	1 (1.2%)
Maternal history		
Chronic hypertension	1 (2.1%)	0
Pre‐pregnancy diabetes	2 (4.2%)	1 (1.2%)
Chronic kidney disease	1 (2.1%)	0
SLE	0	0
Antiphospholipid syndrome	0	3 (3.5%)
Ethnicity		
White	40 (83.3%)	78 (90.7%)
Black	4 (8.3%)	2 (2.3%)
Asian	1 (2.1%)	0
Southeast Asian	3 (6.2%)	6 (7%)
Other	0	0
Mode of conception		
Spontaneous	47 (97.9%)	82 (95.3%)
IVF	1 (2.1%)	3 (3.5%)
Insemination	0	1 (1.2%)
Gestational age (weeks) at diagnosis	26.5 (24.0–29.0)	25.6 (23.0–29.3)
UA PI >95th centile	0	11 (12.8%)
UtA PI >95th centile	0	58 (67.4%)
EFW <3rd centile	0	44 (51.1%)
Pre‐eclampsia at diagnosis	0	0
sFlt‐1/PlGF	3.95 (2.11–7.53)	7.80 (2.89–28.9)
<38	48 (100%)	67 (77.9%)
>85	0	15 (17.4%)
>110	0	13 (15.1%)
>201	0	8 (9.3%)
>655	0	6 (7.0%)
Mean uterine artery Doppler >95th centile	0	58 (67.4%)

*Note:* Continuous data are given as median and interquartile range; categorical data are given as frequency and percentage.

Abbreviations: BMI, body mass index; EFW, estimated fetal weight; FGR, fetal growth restriction; IVF, in vitro fertilisation; PlGF, placental growth factor; sFlt‐1, soluble fms‐like tyrosine‐kinase‐1; SGA, small for gestational age; SLE, systemic lupus erythematosus; UA PI, umbilical artery pulsatility index; UtA PI, mean uterine artery pulsatility index.

**TABLE 2:** Pregnancy outcomes of the study population

	SGA (*n* = 48)	FGR (*n* = 86)
Neonatal weight (g)	2607.5 (2215.0–2941.2)	2160.0 (1650.0–2555.0)
GA at delivery (weeks)	38.0 (37.0–39.0)	37.0 (34.2–37.0)
Delivery <37 weeks	5 (10.4%)	34 (39.5%)
Delivery <34 weeks	1 (2.1%)	16 (18.6%)
Delivery <30 weeks	0	7 (8.1%)
Days from diagnosis to delivery	63.0 (47.2–75.5)	61.0 (41.25–87.5)
Adverse pregnancy outcomes		
Placental abruption	1 (2.1%)	4 (4.6%)
Pre‐eclampsia	1 (2.1%)	17 (19.8%)
Mild	1 (2.1%)	4 (4.65%)
Severe	0	13 (15.1%)
HELLP syndrome	0	2 (2.3%)
Eclampsia	0	1 (1.2%)
Stillbirth	0	2 (2.3%)
Caesarean for non‐reassuring CTG	3 (6.2%)	19 (22.1%)
NICU admission of ≥48 h	9 (18.7%)	36 (41.9%)
Days in NICU	18.5 (7.25–48.5)	17.0 (9.2–39.0)
Neonatal death	0	2 (2.3%)
RDS	3 (6.2%)	16 (18.6%)
BPD	0	7 (8.1%)
Sepsis	1 (2.1%)	3 (3.5%)
Retinopathy (stages III–IV)	0	0
NEC	1 (2.1%)	2 (2.3%)
Intraventricular haemorrhage grade III or IV	1 (2.1%)	0
Periventricular leukomalacia	0	0
Apgar score of <7 at 5 minutes	2 (4.2%)	11 (12.8%)
Artery pH of ≤7.0	0	3 (3.5%)
SGA/FGR severity at delivery		
SGA	42 (87.5%)	0
Antegrade UA flow	5 (10.4%)	76 (88.4%)
UA AEDF	1 (2.1%)	6 (7.0%)
UA REDF	0	4 (4.6%)
Elective delivery	41 (85.4%)	67 (77.9%)
Pre‐eclampsia	0	8 (9.3%)
Fetal Doppler	1 (2.1%)	9 (10.5%)
CTG	1 (2.1%)	5 (5.8%)
Abruption	1 (2.1%)	3 (3.5%)
Stillbirth	0	2 (2.3%)
EFW	30 (62.5%)	33 (38.4%)
Other	8 (16.7%)	7 (8.1%)

*Note:* Continuous data are given as median and interquartile range; categorical data are given as frequency and percentage.

Abbreviations: BPD, bronchopulmonary dysplasia; CTG, cardiotocography; EFW, estimated fetal weight; NEC, necrotising enterocolitis; NICU, neonatal intensive care unit; OBICU, obstetric intensive care unit; RDS, respiratory distress syndrome; TOP, termination of pregnancy.

**FIGURE 1:**
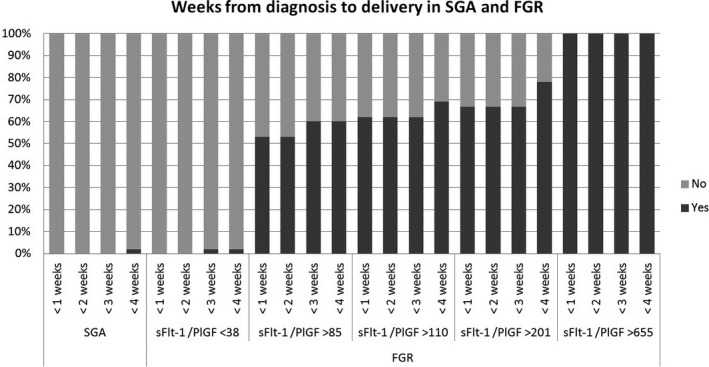
Bar chart showing the frequencies of SGA and FGR cases with delivery at <1, <2, <3 and <4 weeks after diagnosis, according to soluble fms‐like tyrosine kinase‐1/placental growth factor value at diagnosis. FGR, fetal growth restriction; PlGF, placental growth factor; sFlt‐1, soluble fms‐like tyrosinekinase‐1; SGA, small for gestational age

In the SGA cohort, the median interval between diagnosis and delivery was 63.0 days (IQR 47.2–75.5 days). All women with a fetus recorded as SGA had an sFlt‐1/PlGF ratio of below 38 at diagnosis and the time to delivery was longer than 3 weeks in all cases. Thus, sFlt‐1/PlGF < 38 at diagnosis had an NPV of 100% for the need for elective delivery before 3 weeks. More details are presented in Figure 1.

**FIGURE 2:**
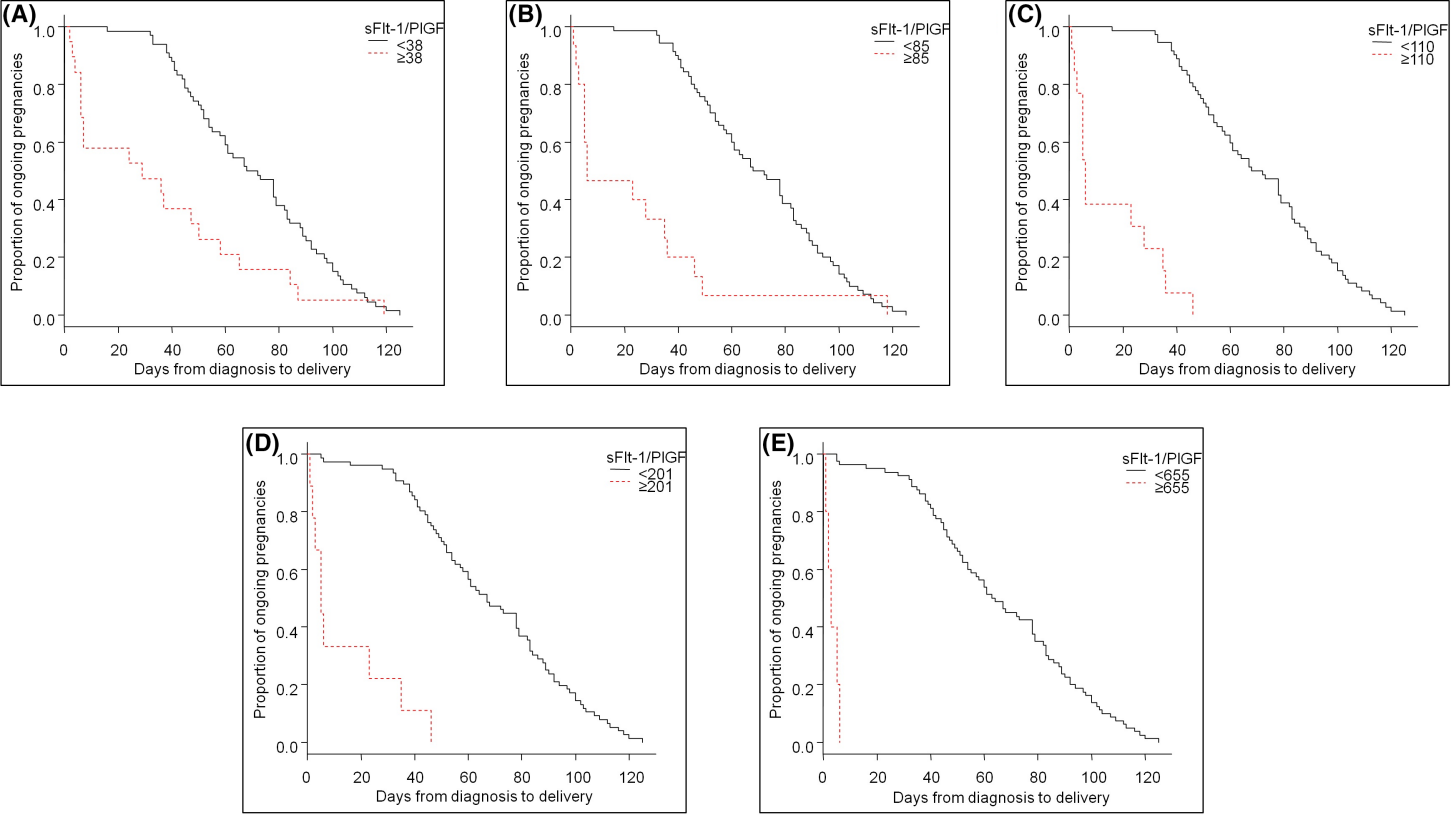
Kaplan–Meier graphs showing time from diagnosis to delivery in pregnancies with early‐onset fetal growth restriction, according to soluble fms‐like tyrosine kinase‐1/placental growth factor values of <38 or ≥38 (A), ≤85 or >85 (B), ≤110 or >110 (C), ≤201 or >201 (D) and ≤655 or >655 (E), at diagnosis

In the FGR cohort, at diagnosis 67 women (77.9%) had an sFlt‐1/PlGF value of <38, four (4.7%) had an sFlt‐1/PlGF value of 38–85, 15 (17.4%) had an sFlt‐1/PlGF value of >85, 13 (15.1%) had an sFlt‐1/PlGF value of >110, eight (9.3%) had an sFlt‐1/PlGF value of >201 and six (7.2%) had an sFlt‐1/PlGF value of >655. The overall median interval to delivery was 61.0 days (IQR 41.2–87.5 days). The interval to delivery was analysed by sFlt‐1/PlGF groups and was significantly shorter in cases with higher sFlt‐1/PlGF values. The median (IQR) intervals were: 70.0 (48.5–91.5) and 28.0 (5.0–53.0) days in cases with sFlt‐1/PlGF values of <38 or ≥38, respectively (*p* < 0.001); 70.0 (50.3–89.8) and 6.0 (5.0–35.5) days in cases with sFlt‐1/PlGF values of ≤85 or >85, respectively (*p* < 0.001); 70.0 (49.8–90.5) and 6.0 (5.0–28.0) days in cases with sFlt‐1/PlGF values of ≤110 or >110, respectively (*p* < 0.001); 67.0 (46.8–89.3) and 5.0 (2.9–23.0) days in cases with sFlt‐1/PlGF values of ≤201 or >201, respectively (*p* < 0.001); and 63.5 (45.0–89.0) and 2.9 (2.0–5.0) days in cases with sFlt‐1/PlGF values of ≤655 or >655, respectively (*p* < 0.001). Kaplan–Meier curves are presented in Figure 2. In the group with an sFlt‐1/PlGF value of <38, there was no need for elective delivery before 2 weeks in any case, and one case (1.5%) required an elective delivery before 4 weeks. A sFlt‐1/PlGF cut‐off value of 38 at diagnosis had an NPV of 100% for the need for elective delivery before 2 weeks and an NPV of 98% for delivery before 4 weeks. By contrast, more than 50% of cases with sFlt‐1/PlGF >85 required elective delivery before 1 week, rising to 100% in cases with sFlt‐1/PlGF >655. More details are presented in Table 3 and Figure 1.

**TABLE 3:** Sensitivity, specificity, positive predictive value and negative predictive value of different soluble fms‐like tyrosine kinase‐1/placental growth factor cut‐off values used to predict delivery at <1, <2, <3 and <4 weeks

sFlt‐1/PlGF	Time to delivery	FGR with antegrade umbilical artery flow (*n* = 86)			
		Sn (95% CI)	Sp (95% CI)	PPV (95% CI)	NPV (95% CI)
38 (*n* = 67)	<1 week	1 (0.52–1)	0.86 (0.76–0.93)	0.42 (0.2–0.66)	1 (0.92–1)
	<2 weeks	1 (0.52–1)	0.86 (0.76–0.93)	0.42 (0.2–0.66)	1 (0.92–1)
	<3 weeks	0.89 (0.52–1)	0.86 (0.76–0.93)	0.42 (0.2–0.66)	0.98 (0.92–1)
	<4 weeks	0.9 (0.55–1)	0.87 (0.77–0.93)	0.47 (0.24–0.71)	0.98 (0.92–1)
85 (*n* = 15)	<1 week	1 (0.52–1)	0.9 (0.82–0.96)	0.53 (0.27–0.79)	1 (0.92–1)
	<2 weeks	0.89 (0.52–1)	0.91 (0.82–0.96)	0.53 (0.27–0.79)	0.99 (0.92–1)
	<3 weeks	0.9 (0.55–1)	0.92 (0.84–0.97)	0.6 (0.32–0.84)	0.99 (0.92–1)
	<4 weeks	0.9 (0.55–1)	0.92 (0.84–0.97)	0.6 (0.32–0.84)	0.99 (0.92–1)
110 (*n* = 13)	<1 week	1 (0.52–1)	0.94 (0.86–0.98)	0.61 (0.32–0.86)	1 (0.93–1)
	<2 weeks	1 (0.52–1)	0.94 (0.86–0.98)	0.61 (0.32–0.86)	1 (0.93–1)
	<3 weeks	1 (0.52–1)	0.94 (0.86–0.98)	0.61 (0.32–0.86)	1 (0.93–1)
	<4 weeks	0.9 (0.55–1)	0.95 (0.87–0.98)	0.69 (0.39–0.9)	0.99 (0.93–1)
201 (*n* = 8)	<1 week	0.75 (0.35–0.97)	0.97 (0.91–1)	0.75 (0.35–0.97)	0.97 (0.91–1)
	<2 weeks	0.75 (0.35–0.97)	0.97 (0.91–1)	0.75 (0.35–0.97)	0.97 (0.91–1)
	<3 weeks	0.67 (0.3–0.92)	0.97 (0.9–1)	0.75 (0.35–0.97)	0.96 (0.89–0.99)
	<4 weeks	0.6 (0.26–0.88)	0.97 (0.9–1)	0.75 (0.35–0.97)	0.95 (0.87–0.99)
655 (*n* = 6)	<1 week	0.5 (0.07–0.93)	1 (0.93–1)	1 (0.09–1)	0.97 (0.91–1)
	<2 weeks	0.5 (0.07–0.93)	1 (0.93–1)	1 (0.09–1)	0.97 (0.91–1)
	<3 weeks	0.4 (0.05–0.85)	1 (0.93–1)	1 (0.09–1)	0.96 (0.89–0.99)
	<4 weeks	0.33 (0.04–0.77)	1 (0.93–1)	1 (0.09–1)	0.95 (0.88–0.99)

Abbreviations: CI, confidence interval; NPV, negative predictive value; PlGF, placental growth factor; PPV, positive predictive value; sFlt‐1, soluble fms‐like tyrosine‐kinase‐1; Sn, sensitivity; Sp, specificity.

## 4 DISCUSSION

### 4.1 Main findings

This study provides evidence that the sFlt‐1/PlGF ratio can be useful for classifying the severity of FGR and to assess the interval to delivery among cases with similar baseline severity and prognosis, regarding Doppler and ultrasound findings. An sFlt‐1/PlGF ratio cut‐off value of 38 has an NPV of 100% for ruling out the need for elective delivery before 3 weeks for fetuses that are SGA and before 2 weeks for fetuses with FGR. By contrast, the sFlt‐1/PlGF cut‐off value of 85 is associated with a shorter time to delivery, requiring an elective delivery before 1 week in more than 50% of cases.

### 4.2 Strengths and limitations

The main strengths of this study are its prospective design in a relatively large cohort of early‐onset FGR and SGA. Additionally, this study provides valuable information that could be used for tailoring the frequency of fetal scans based on sFlt‐1/PlGF values. In Spain, most centres have the sFlt‐1/PlGF value available for use in clinical practice for women with suspected PE. According to the PROGNOSIS trial protocol,^10^ FGR is considered a PE‐related finding; therefore, the determination of sFlt‐1/PlGF is indicated in such cases.

We acknowledge some limitations of this study. First, computerised CTG was not available at our centre; thus, our results may not be applicable to cases with such a tool available for fetal monitoring. Second, the sFlt‐1/PlGF results were not blinded to the investigators, which could have influenced the fetal surveillance protocol; nevertheless, the indication for delivery was based strictly on ultrasound and CTG findings in all cases. Third, chromosomal anomalies and congenital defects were excluded in this study; thus, our results are not applicable to such cases. Fourth, our results might be of great value for tailoring follow‐up and prenatal counselling; however, they cannot be used for planning delivery. Finally, only 15 cases had an sFlt‐1/PlGF value of >85, which could limit the external validity of our findings.

### 4.3 Interpretation

Previous studies have shown that high sFlt‐1/PlGF values in FGR and PE reveal an antiangiogenic status, which is caused by the underlying placental dysfunction.^22^ The sFlt‐1/PlGF ratio has demonstrated that it is useful for predicting pregnancy complications associated with placental insufficiency, and particularly for ruling out PE in pregnancies with FGR or other clinical signs and symptoms of PE.^10^ Recently, several studies have shown that the sFlt‐1/PlGF ratio values in early‐onset FGR rise as Doppler severity increases, which leads to shorter times to delivery, lower birthweight *z*‐scores and greater risks of adverse perinatal outcomes.^12^, ^23^ Another study in a cohort of 122 cases of early‐onset FGR with antegrade UA flow showed that 35.6% of cases with sFlt‐1/PlGF values of >85 and none of the cases with sFlt‐1/PlGF values of ≤85 required an elective delivery within 1 week.^11^ These findings are similar to our findings here; however, in that study no other cut‐off values were investigated, and nor were the NPV and PPV values used for predicting and excluding the need for elective delivery in subsequent weeks.

Predicting the time between diagnosis and delivery in SGA and FGR fetuses is challenging, as the progression of Doppler deterioration can vary widely;^24^ thereby, it is difficult to establish from Doppler findings alone the optimal interval between ultrasounds in the management of these fetuses. In early‐onset cases with antegrade flow in UA, management recommendations vary considerably as there is no consensus on the adequate frequency of scans;^7^ nevertheless, most international guidelines advise that Doppler ultrasound should be performed at least weekly for FGR and every 2 weeks for fetuses that are SGA.^7^ In this study we show that sFlt‐1/PlGF values could be useful for individualising fetal surveillance, as they have shown to be highly associated with the interval to delivery.

For these reasons, our study has important clinical implications as it has shown excellent performance for detecting pregnancies at a higher risk of fetal distress, which require more frequent scans, but also cases at a lower risk of complications that could benefit from a less strict surveillance protocol. According to our results, FGR cases with sFlt‐1/PlGF values of >85 should be closely monitored for the early detection of fetal or maternal complications. By contrast, in pregnancies with a sFlt‐1/PlGF value of <38 it might be possible to extend the interval between scans to up to 3 weeks for SGA and up to 2 weeks for FGR with antegrade UA flow, which would significantly reduce the number of fetal ultrasounds, thereby lowering parental anxiety and the burden on the healthcare system.

## 5 CONCLUSION

sFlt‐1/PlGF values in early‐onset SGA and FGR are predictive of the time to delivery and could be used for planning fetal surveillance by reducing the frequency of ultrasounds in cases with sFlt‐1/PlGF < 38 and by providing closer follow up in cases with sFlt‐1/PlGF >85.

## CONFLICT OF INTEREST

Manel Mendoza has received lecture fees from Roche Diagnostics. The other authors report that they have no conflicts of interest associated with this work. Completed disclosure of interests form available to view online as supporting information.

## AUTHOR CONTRIBUTIONS

EB, MM, PG‐M and BS had full access to all of the data in the study and take responsibility for the integrity of the data and the accuracy of the data analysis. EB and MM conceived and designed the study. PG‐M, BS, AF, IH, RF‐O, LI and MA‐A contributed to the literature research. EB, MM, AF, PG‐M, BS, LI and MA‐A contributed to data collection and confirmation. EB, MM, PG‐M and BS contributed to data analysis, and EB, MM, PG‐M, BS, AF and EC contributed to data interpretation. EB and MM drafted the article. EC made substantial revisions to the article.

## ETHICAL APPROVAL

The Vall d'Hebron University Hospital Ethics Committee (CEIC‐VHIR PR[AMI]349/2016) approved the study protocol. Informed consent was obtained from all patients, which was included in the patient’s medical record.

## Supporting information


**Appendix** S1. Supplementary Information.

**Appendix** S2. Supplementary Information.

**Appendix** S3. Supplementary Information.

**Appendix** S4. Supplementary Information.

**Appendix** S5. Supplementary Information

**Appendix** S6. Supplementary Information.

**Appendix** S7. Supplementary Information.

**Appendix** S8. Supplementary Information.

**Appendix** S9. Supplementary Information.

**Appendix** S10. Supplementary Information.

## Data Availability

Data available on request from the authors.
